# Quantitative Analyses of Skin Cancer Research in Pakistan

**DOI:** 10.12669/pjms.37.2.2889

**Published:** 2021

**Authors:** Syeda Hajrah Rehman, Bushra Majid, Sofia Ali Syed, Muhammad Asif Qureshi

**Affiliations:** 1 Syeda Hajrah Rehman, MBBS Student. Dow Medical College, Dow University of Health Sciences, Karachi, Pakistan; 2 Bushra Majid, MBBS Student. Dow Medical College, Dow University of Health Sciences, Karachi, Pakistan; 3 Sofia Ali Syed, BDS, M.Phil. Associate Professor, Dow Dental College, Dow University of Health Sciences, Karachi, Pakistan; 4 Muhammad Asif Qureshi, MBBS, PhD, Postdoc (Germany), M.A. Professor of Pathology, Dow International Medical College, Dow University of Health Sciences, Karachi, Pakistan

**Keywords:** Skin cancer, Research output, International literature, Pakistan

## Abstract

In order to investigate the current status of skin cancer research output in Pakistan, *International* (PubMed) and national (PakMediNet) scientific databases were searched using variety of keywords to retrieve relevant publications. A strict inclusion criterion was applied to select skin cancer publications for final analyses. Data were recorded by two authors and consistent data were entered into SPSS and Microsoft Excel and analyzed for annual growth rate and frequencies. Of 116 articles that were finally included in the study, 74 were original articles, 24 were case-reports, 10 were review articles, three were editorials, two were research communications and one each of case-series, correspondence and response to letter to the editor. The first article on skin cancer from Pakistan was published in 1976 whereas the last article included in our study was published in December 2018. Excluding Karachi, most of the cities have no contribution in the field of skin cancer. Since 1976 to date, the average number of publications per year has been low, with only 2.7 publications per year. Skin cancer research is alarmingly scarce in Pakistan. This calls for immediate attention by all concerned to contribute and devise appropriate measures towards skin cancer research in Pakistan.

## INTRODUCTION

Skin cancer is the fifth commonest cancer across the globe accounting for approximately 5.8% of cases (melanoma being 1.6%) and an overall mortality of 0.6 to 0.7 %.^1^ In Pakistan, while there are no national level cancer registration system, regional cancer registries like Punjab Cancer Registry (PCR) and others have reported high incidence of skin cancers in contrast to Globocan 2018 report which lists non melanoma skin cancers (NMSC) as 18^th^ and melanoma as 32^nd^ most common cancer of Pakistan.^2^,^3^ According to the PCR, skin cancer ranks as the eighth and ninth most common cancer in females and males respectively in 2017.^4^ Moreover, Karachi Cancer Registry (KCR) also reported skin cancer amongst the top eight cancers in females in Karachi.^5^ Recently, a pathology-based cancer registry of Dow University of Health Sciences (DUHS) listed NMSC, as the fifth most commonly diagnosed cancer (being second most common in males and fifth most common in females), suggesting a substantial increase of skin cancer burden in Karachi.^6^ Reported cases in Karachi and Larkana are far common than in other parts of Pakistan (like Hazara-district) proving the well-established fact that there exists an inverse relation between skin cancer incidence and distance from the equator.^7^

Taken together, these data suggest an increase of skin cancer throughout Pakistan in recent years.^1^,^6^ It is therefore highly plausible to undertake appropriate measures to address this cancer type. One such step could be to identify if Pakistani scientists are investigating this cancer appropriately to identify novel molecules of pathogenic, diagnostic, therapeutic and prognostic significance. To the best of our knowledge, there are no systematic analyses available to delineate the current status of skin cancer research in Pakistan.

In the research described herein, we quantitatively investigated the current status of skin research output in Pakistan. The objectives of this study were (a) to investigate the total skin cancer research output to date originating from Pakistan, (b) to investigate the types/categories and research foci of skin cancer research related publications published from Pakistan, and (c) to investigate major contributing universities/research centres and cities towards skin cancer research output from Pakistan.

## METHODS

**Fig.1:**
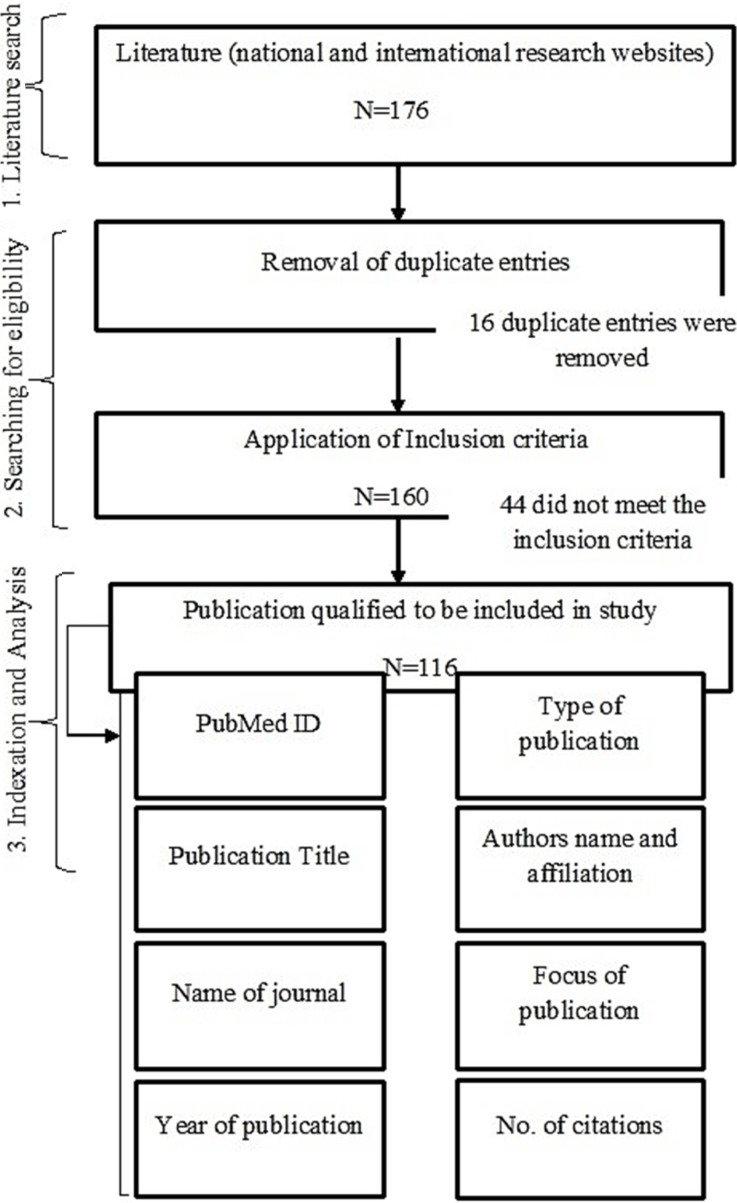
Schematic Representation of the Working-Algorithm of Study.

This observational study was conducted at the Dow University of Health Sciences Karachi using a three-step inclusion criteria stratified approach; 1) Literature-search on skin cancer 2) Shortlisting of publications/studies according to inclusion criteria 3) Indexation and analyses of selected studies (Fig.1). Since this study was based on tertiary analyses of publically available data, it does not require ethical/IRB approval.

All duplicate entries were excluded from analyses. Only those publications were included in the study where a) skin cancers in Pakistan was the primary focus of publication or discussed in other ways such as epidemiological study-designs addressing skin cancer-burden in Pakistan b) studies conducted in Pakistan or their area of concern was skin cancer in Pakistan c) at least first author was affiliated to any institution of Pakistan.

Details of the steps undertaken in the analyses described herein are described as follows; Literature search was performed by two authors. In order to retrieve skin cancer related literature in Pakistan, both international (PubMed) and national (PakMediNet) scientific databases were searched irrespective of study design. In order to extract maximum number of literature search, a wide variety of key words including “skin-malignancy and Pakistan”, “skin cancer and Pakistan”, “squamous-cell-carcinoma and Pakistan”, “skin-neoplasia and Pakistan”, “basal-cell-carcinoma and Pakistan”, “melanoma and Pakistan”, “skin-carcinoma and Pakistan”, “skin-cancer-epidemiology and Pakistan”, “cutaneous-malignancies and Pakistan”, “cutaneous-cancers and Pakistan”, “cutaneous-neoplasia and Pakistan”, “skin metastasis and Pakistan” and “ cutaneous-carcinoma and Pakistan” were entered. To further strengthen maximum number of literature search, Google and Google-Scholar search engines with restriction to retrieve webpages of Pakistan were used. Moreover, we did not use any database filters.

Two authors indexed the data in Microsoft-Excel (MS) sheets separately to avoid any error. As previously described,^8^ the selected studies were recorded for their PubMed ID, year and type of publication, name of the journal, title and design of study, authors affiliation/institution, research focus, and number of citations. Publications which were not indexed in PubMed (Medline) were included in non-indexed publications. The entered data of both the authors were compared and discrepancies (if found) were removed to generate a final indexed data-sheet. Final data were then entered into SPSS to calculate annual growth rate and descriptive analyses as described in Table-I and Table-II. All descriptive statistical analyses described herein were conducted using SPSS.

**Table-I:** Major Journals to publish skin cancer research from Pakistan.

Name of journals*	*No. of publications n (%)*	*PubMed indexed*	*Impact factor*	*H-index*
J Pak Med Assoc	10(8.62%)	Yes	0.718	
Ann Med Surg	7(6.03%)	Yes	-	
Asian Pac J Cancer Prev	7(6.03%)	Yes	-	
J Coll Physicians Surg Pak	6(5.17%)	Yes	0.439	
Skin Res Technol	5(4.31%)	Yes	1.489	
J Pak Assoc Derma	4(3.44%)	No	-	
J Cutan Pathol	3(2.58%)	Yes	-	
Pak Armed Forces Med J	3(2.58%)	No	-	

* journals with more than 2 publications are included

**Table-II:** Major Contributing Institutions to Skin Cancer Research in Pakistan.

*University/Research Institutions*	*City*	*N (%)*
Agha Khan University Hospital	Karachi	11(9.48%)
Dow University of Health Sciences	Karachi	7(6.03%)
Armed Forces Institute of Pathology	Rawalpindi	5(4.31%)
Pakistan Institute of Medical Sciences	Islamabad	5(4.31%)
Quaid-e-Azam University	Islamabad	5(4.31%)
King Edward Medical University	Lahore	4(3.44%)
National Textile University	Faisalabad	4(3.44%)
COMSATS Institute of Information Technology	Wah	3(2.58%)
Institute of Radiotherapy and Nuclear Medicine (IRNUM)	Peshawar	3(2.58%)
Sindh Institute of Urology and Transplantation (SIUT)	Karachi	3(2.58%)

## RESULTS

Initially, a total of 176 studies were retrieved. Of these, 16 duplicate entries were removed leaving a total of 160 studies were further analysed. Of 160 studies, 44 did not meet the inclusion criteria, and were removed from our study. The remaining 116 studies were subjected to final analyses described herein (Fig.1).

### Annual growth of publications

**Fig.2:**
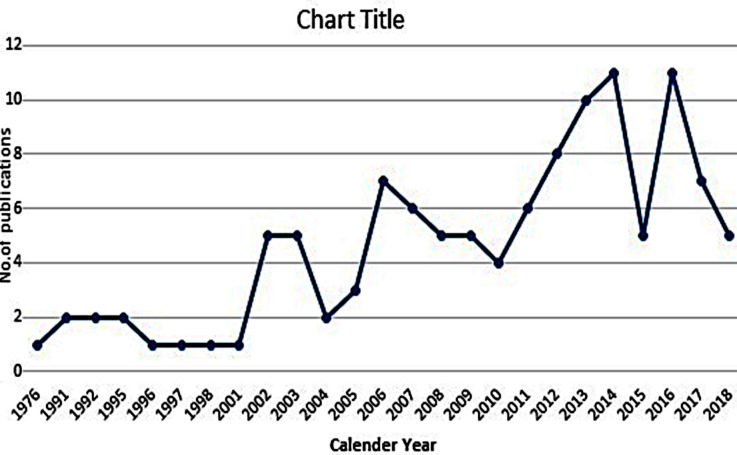
Annual Growth of Skin Cancer Related Publications from Pakistan since 1976 to date.

The first and last article on skin cancer from Pakistan was published in 1976 and 2018 respectively. Only 10 studies were published during 24 years since the first publication. A total of 16 articles were published during 2000-2005 (annual growth rate of 9.96%). Within the next five years (2006-2010), 26 articles were published (annual growth rate of 12.5%). During 2011-2015, a total of 40 articles were published (annual growth rate of 10.76%). From 2016 to 2018, 24 articles were published (annual growth rate of -13.3%) with 2016 being the year with highest number of published articles (n=12). Since 1976 to date, the average number of publications per year has been low, with only 2.7 publications per year (Fig.2)

### Types and research foci of publications

Of the 116 articles, 81% (n=94) were published in indexed (PubMed) journals and 19% (n=22) were published in non-indexed journals. Table-I represents the major journals (journals with more than two publications were listed) which published the skin cancer research from Pakistan. Of the 116 publications, 63.7% (n=74) were original-articles, 20.6% (n=24) were case-reports, 8.6% (n=10) were review-articles, three were editorials, two were research-communications and one each of case-series, correspondence and response to letter to the editor. Of the 74 original-articles, 28.3% (n=21) were based on the prevalence and occurrence of the skin cancer, 14.8% (n=11) publications were hospital based, 8.1% (n=6) were pathology based, four population based, 14.8% (n=11) were centred on the diagnostic modalities for skin cancer, 10.8% (n=8) studies were laboratory-based, 12.2% (n=9) focused on therapeutic-interventions for various skin malignancies and 2.7% (n=2) were comparisons. Of 24 case-reports, 25% (n=6) comprised benign yet unique presentations of cutaneous-tumours while 75% (n=18) discussed the malignant-tumours presenting at a rare site or as a rare occurrence while one case reported skin malignancy as part of Gorlin-syndrome.

Of the 10 reviews, 40% (n=4) discussed the aetiology and diagnosis of skin malignancies, 20% (n=2) focused on the role of anticancer agents for various malignancies including the skin cancer and 20% (n=2) focused on the management, 10% (n=1) focused on the protective factor for skin cancer while one reviewed the mechanism of melanomagenesis.

Of two research-communications, one compared the clinical features/outcome of patients with nodal or extra-nodal non-Hodgkin-lymphoma while the other one focused on epidemiology of basal-cell-carcinoma and importance of clearing excision margins of the malignancy. The three editorials highlighted the importance of cancer registration, case of nodular-melanoma and its management and role of flavonoid in skin cancer.

The case-series focused on malignant-cutaneous-adnexal-tumour while the correspondence focused on various features of melanocytic-nevi across diverse geographical locations.

### Authors affiliations and contributing institutions

A total of 54 Pakistani institutions/research centres contributed in publications. Of these, 67% (n=34) were from public/government set-up, 29.6% (n=16) were from private setups. Leading institute was Agha Khan University Hospital (AKUH), Karachi. Interestingly, these 54 institutions were only from the 19 cities of Pakistan with Karachi being the main contributor. The analysis thus revealed that most of the cities of Pakistan have no contribution in the field of skin cancer (Table-II)

### Number of citations received

Of 116 articles, a total of 13.7% (n=16) articles did not receive any citation. Of these, 25% (n=4) and 75% (n=12) articles were published in indexed and non-indexed journals respectively. The remaining 100 articles were cited at least once and therefore summed-up to a total of 1886 citations. Of these, 77.4% (n=1460) citations belonged to the original-articles under various sub-categories namely therapeutic, diagnostic, genetics, hospital/laboratory, histopathology, clinicopathological and comparison based. Of 1460 citations, a total of 30% (n=432) citations were received by laboratory-based studies which by large showed the maximum number of citations in skin cancer research.

## DISCUSSION

With a substantial rise in skin cancer burden in Pakistan, it is highly plausible to investigate if the rise is paralleled by relevant research. This report, to the best of our knowledge is the first report that systematically present all data available with reference to skin cancers in Pakistan. We report extreme paucity of skin cancer research in Pakistan reflecting a generalized neglect towards healthcare research as also highlighted in the National Health Plan.^9^

Most of the studies were related to frequency of skin cancer at regional level with paucity of national level data. Moreover, only 2.6 publications/year indicates skin cancer research in Pakistan as an un-attended issue. Alarmingly, majority of the skin cancer research published from Pakistan was not published in indexed journals, leading to low visibility and citation of these reports.^10^,^11^

Of 116 studies that we investigated, only 74 were original-articles while 21 of them were based on the prevalence/frequencies of skin cancer. Only three studies discussed post-transplant skin malignancies which are documented as common malignancies and common cause of mortality in transplant recipients.^12^ Sindh Institute of Urology and Transplantation (SIUT), being one of the major transplant centres in Pakistan contributed only two studies related to post-transplant malignancies. The third was a review published by DUHS.

Alarmingly, little investigation has been made to address novel diagnostic/treatment modalities regarding skin cancer research in Pakistan. We did not find any relevant randomized-controlled-trials or meta-analysis in this regard. These facts highlight obvious lack of priority to skin cancer research by the researchers as well as relevant policy makers.

Highest number of publications were published from Karachi followed by Islamabad. Private institute with the largest number of publications since 1976 was AKUH with 11 published studies. Of these, four were reviews, three case-reports and three original-articles. No article was published from this institute on skin cancer in 2018. Such little output by a 1203 bedded-hospital is a matter of great concern.^13^ A public-sector university (DUHS) contributed total seven studies. Of these, three were case-reports, three were original-articles and one was review-article. However, no article was published for last two years. Lack of funds, research guidelines and proper statistics of skin cancer in the country may have allowed this situation to occur. Moreover, SKMH&RC (since inception) produced only two publications-an evidence of negligible performance by its research department.

It is important to mention that Pakistan shares a functionally active border with neighbouring countries (such as Iran and Afghanistan) where skin cancer incidence is high.^1^ There was only one study from Pakistan describing skin cancer as the 2^nd^ most common malignancy in Afghan refugee population.^14^ Since then, number of refugees, sociodemographics and cultural practices have changed. It is therefore highly relevant for researchers in those areas to conduct follow up studies to delineate current situation of skin cancer in those areas. These data suggest the dire need for setting-up a national cancer registry that can ease the direction of decision-makers and future researchers.^15^ Taken together, we highlight serious paucity of skin cancer research in Pakistan requiring immediate attention by all relevant stakeholders.

### Limitations of the study

While the study effectively highlights status of skin cancer research in Pakistan, it is, by definition of the inclusion criteria described herein, unavoidably possible that articles are not listed in PubMed and/or PakMediNet are missed in the analyses described herein.

## CONCLUSION

Skin cancer research is alarmingly scarce in Pakistan. This calls for immediate attention by health policy-makers, institutions as well as researchers to contribute and devise appropriate measures towards skin cancer research in Pakistan.

### Author’s Contribution:

**SHR:** Data retrieval, indexation, and manuscript drafting.

**BM:** Data retrieval, indexation and manuscript drafting.

**SAS**: Facilitation and monitoring of data analyses and manuscript drafting.

**MAQ:** inception of idea, execution and management of the project. Data indexation, supervision of data analyses and is responsible for integrity of results described herein.

All authors read and agreed to the final version of the manuscript.
